# Female Choice for Males with Greater Fertilization Success in the Swedish Moor Frog, *Rana arvalis*

**DOI:** 10.1371/journal.pone.0013634

**Published:** 2010-10-26

**Authors:** Craig D. H. Sherman, Jörgen Sagvik, Mats Olsson

**Affiliations:** 1 Centre for Integrative Ecology, School of Life and Environmental Sciences, Deakin University, Waurn Ponds, Victoria, Australia; 2 School of Biological Sciences, The University of Wollongong, Wollongong, New South Wales, Australia; 3 Department of Zoology, Göteborg University, Göteborg, Sweden; 4 School of Biological Sciences, The University of Sydney, Sydney, New South Wales, Australia; Queens University, Canada

## Abstract

**Background:**

Studies of mate choice in anuran amphibians have shown female preference for a wide range of male traits despite females gaining no direct resources from males (i.e. non-resource based mating system). Nevertheless, theoretical and empirical studies have shown that females may still gain indirect genetic benefits from choosing males of higher genetic quality and thereby increase their reproductive success.

**Methodology/Principal Findings:**

We investigated two components of sexual selection in the Moor frog (*Rana arvalis*), pre-copulatory female choice between two males of different size (‘large’ vs. ‘small’), and their fertilization success in sperm competition and in isolation. Females' showed no significant preference for male size (13 small and six large male preferences) but associated preferentially with the male that subsequently was the most successful at fertilizing her eggs in isolation. Siring success of males in competitive fertilizations was unrelated to genetic similarity with the female and we detected no effect of sperm viability on fertilization success. There was, however, a strong positive association between a male's innate fertilization ability with a female and his siring success in sperm competition. We also detected a strong negative effect of a male's thumb length on his competitive siring success.

**Conclusions/Significance:**

Our results show that females show no preference for male size but are still able to choose males which have greater fertilization success. Genetic similarity and differences in the proportion of viable sperm within a males ejaculate do not appear to affect siring success. These results could be explained through pre- and/or postcopulatory choice for genetic benefits and suggest that females are able to perceive the genetic quality of males, possibly basing their choice on multiple phenotypic male traits.

## Introduction

When Andersson reviewed the field of sexual selection in animal and plants more than 15 years ago, female mate choice had already been confirmed in more than 150 taxa on more than 30 male traits [1]. These demonstrations represent underlying selection, directly or indirectly, on virtually every sensory system, from vision and hearing, to smell and perception of vibrations. Nevertheless, despite the plethora of demonstrations of female mate choice, we are sometimes still surprised at females' ability to detect subtle male traits and fine-tune their investment patterns into fitness benefits. For example, experimental reduction of male attractiveness via manipulation of UV-reflecting traits in European Blue tits (*Parus major*) resulted in females associating with manipulated males overproduce daughters [2]. Similarly, when females of the polymorphic Gouldian finch (*Erythrura gouldiae*) are presented with genetically more incompatible partners of alternative morphs, they overproduce the homozygous sons that will limit production of genetically compromised heterogametic daughters [3]. Thus, females can base their choice of potential mates on subtle male characteristics and there is clearly still much to be learned about female decision making.

Studies of mate choice in anuran amphibians have shown female preference for a wide range of male acoustic and morphological traits. Females of many species often show a preference for call properties that involve higher energy expenditure, with males with faster call rates, higher call intensities and low frequencies calls having greater mating success [4], [5], [6], [7]. Morphological traits such as body size and condition, nuptial pad size and colouration have also been shown to be important in mate selection [8], [9], [10]. Females however, may use multiple traits that convey information on different aspects of a male's fitness or gives an indication of male fitness over a range of traits [11], [12]. For example, Taylor et al. [11] showed that female squirrel tree frogs, *Hyla squirella*, show a strong preference for both call frequency and males with a large lateral body stripe, while female Túngara frogs (*Physalaemus pustulosus*) show a preference for male calls that are accompanied by a video playback of a calling male with vocal sac inflation [13]. Thus, females may base their choice on multiple complex traits, but few studies have shown if this preference leads to an increase in reproductive success and/or offspring fitness [8].

An increasing number of studies have shown that polyandry and sperm competition are much more prevalent among amphibians than previously thought [14], [15], [16], [17], [18], [19], [20]. Females are thought to gain indirect genetic benefits from polyandry and a number of studies have shown both good genes and genetic compatibility effects on fertilization success and offspring fitness [14], [18], [21], [22]. For example, in the polyandrous quacking frog, *Crinia georgiana*, studies have shown significant male × female interaction effects on fertilization success and offspring fitness such as embryo survival and survival to metamorphosis [18], while in the Australian tree frog, *Litoria peronii*, both genetic relatedness and intrinsic male quality can influence fertilization success in sperm competition [21], [22]. Thus the reproductive success of males and females are not only influenced by choices made before copulation, but also by processes operating after copulation such as sperm competition and gamete recognition systems.

The Moor frog *Rana arvalis* is an explosive breeder forming large leks during the spring breeding season [23], [24]. As in many amphibians, the operational sex ratio on any given night is highly skewed towards males, with genetic analysis of natural clutches revealing multiple paternity in 14% to 29% of clutches [20]. This suggests that sperm competition, through multiple mating or sperm leakage, plays an important role in the mating system of this species. The current study set out to test the importance of a number of male traits on female mate choice and subsequent *in vitro* fertilization success in *R. arvalis*. Firstly, we tested pre-copulatory female choice for males of two different size classes. Secondly, our recent research on other amphibians have demonstrated effects of genetic similarity on fertilization success in sperm competition [22], and male innate fertilization ability [21]. Therefore, we also include these post-copulatory aspects in our analysis of female mate choice, along with male arm length and thumb length which are known to be sexually selected traits in male-male competition and male-female amplexus [10], [25].

## Results

### Female pre-copulatory mate choice

Thirteen females showed a preference for the smaller male while the remaining six showed a preference for the larger male, however this trend was not statistically significant (one tailed binomial test *P* = 0.084).Our logistic regression showed that the only significant predictor of female mate choice was intrinsic male siring success in controls (log likelihood ratio test, *X* ^2^ = 13.0, *P*<0.001, *df*  = 1), followed by a non-significant difference in thumb length (*X* ^2^ = 3.4, *P* = 0.07, *df*  = 1).

### Male siring success in sperm competition

**Figure 1:**
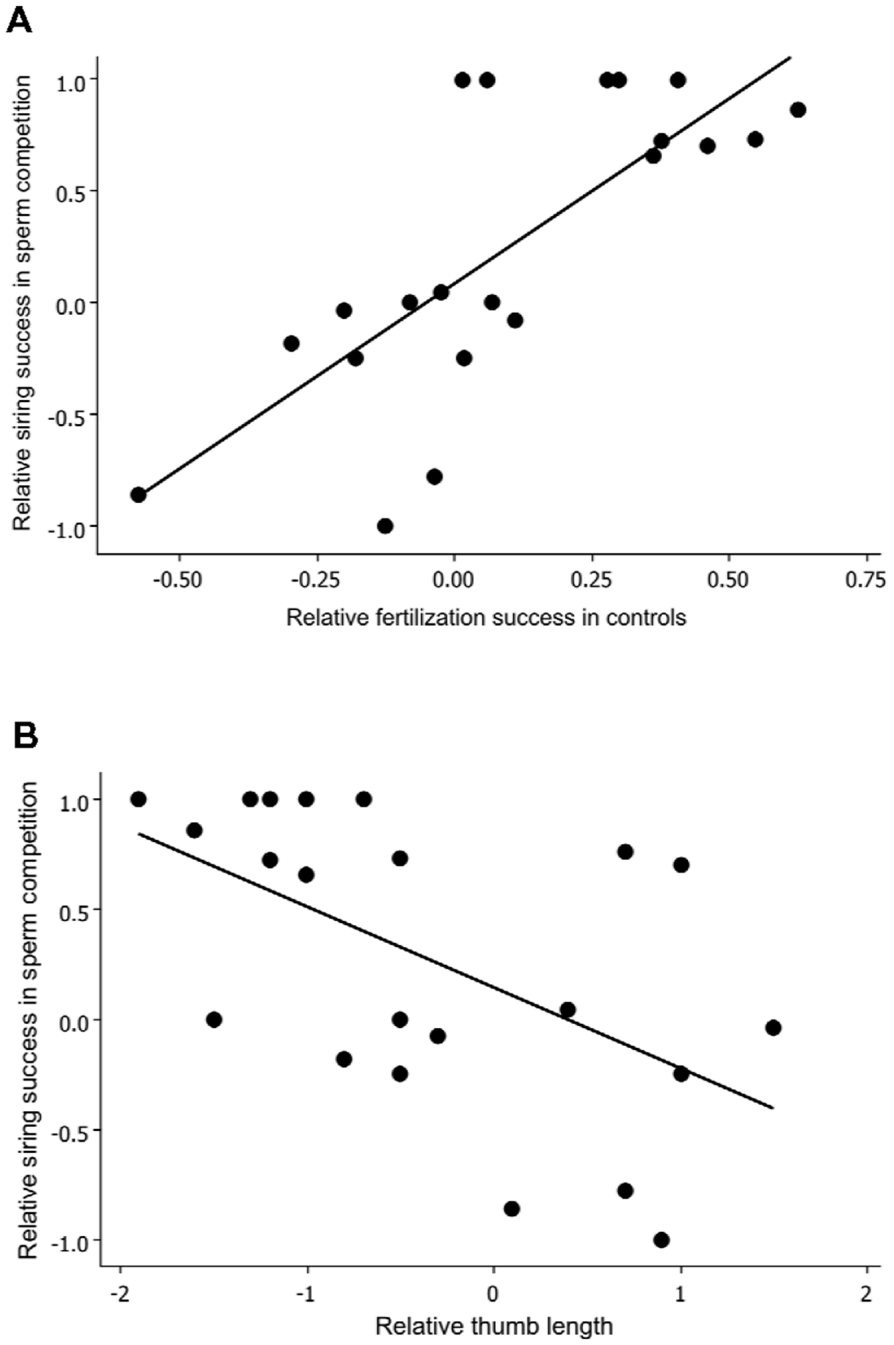
The relationship between the differential siring success of males in sperm competition and (A) their differential fertilization success in controls (*F*_2, 20_  = 21.2, *P* = 0.0002) and (B) their differential thumb length (*F*_2, 20_  = 9.30, *P* = 0.007).

Relative siring success of males in competitive fertilizations ranged from 0–100% (mean  = 51%±6% SE), while intrinsic fertilization success in the controls varied from <1% up to 100% (mean  = 57%±5% SE). The multiple regression analysis explained 71% of the variation in the difference in siring success between the two males (final model: r^2^  = 0.71, *F* = 22.6, *P*<0.0001) with the only two remaining, significant predictors (after backward elimination) being the difference in innate fertilization success (control fertilizations) (*β* = 1.36, ±0.29 SE, *F*_2, 20_  = 21.2, *P* = 0.0002; Figure 1A) and the difference in thumb length (*β* = −0.28, ±0.09 SE, *F*_2, 20_  = 9.30, *P* = 0.007; Figure 1B).The difference in body mass was non-significant (*P* = 0.87), and we therefore did not control for body mass in the final analysis (residuals from a thumb length – snout-vent length regression was equally significant as thumb length itself). These predictors remain significant when controlling for multiple tests (sequential Bonferroni) [26].

### Hatching success

**Figure 2:**
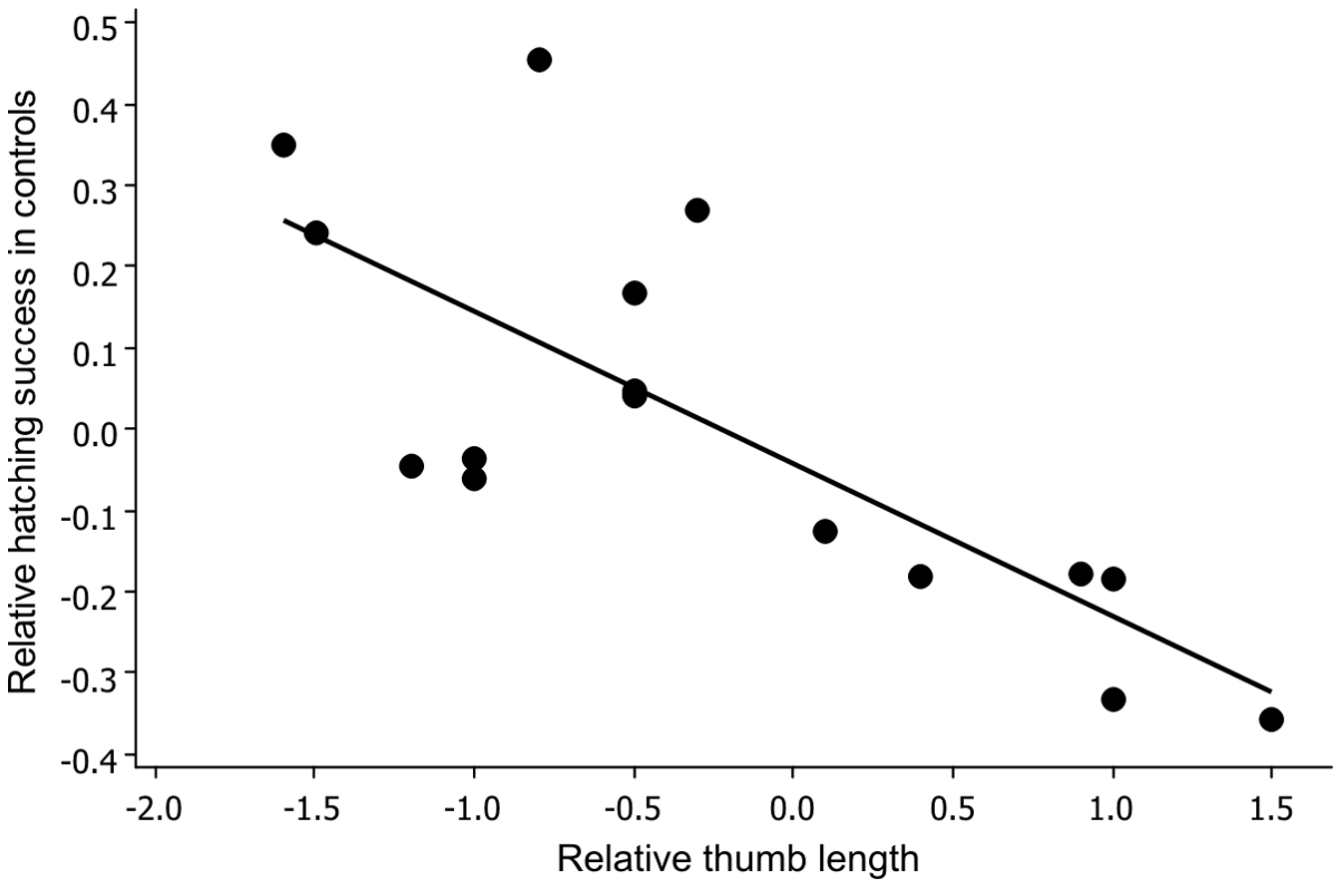
Relationship between the differential hatching success of males in controls and their differential thumb length (*F*_2, 20_  = 17.7, *P* = 0.0012).

Overall hatching success of fertilized eggs was high with on average 82% (±3% SE) of fertilized eggs hatching. Relative hatching success in controls was only significantly predicted by the difference in thumb length between males (*β* = −0.20, ±0.05 SE, final model: *R* ^2^ = 0.61, *F*_2, 20_  = 17.7, *P* = 0.0012; Figure 2). Neither difference in fertilization success (*F* = 1.06, *P* = 0.32) nor male size (*F* = 0.34, *P* = 0.57) were significant predictors in this multiple regression. This trait is significant when controlling for multiple tests (sequential Bonferroni) [26].

### Male traits and sperm quality

**Figure 3:**
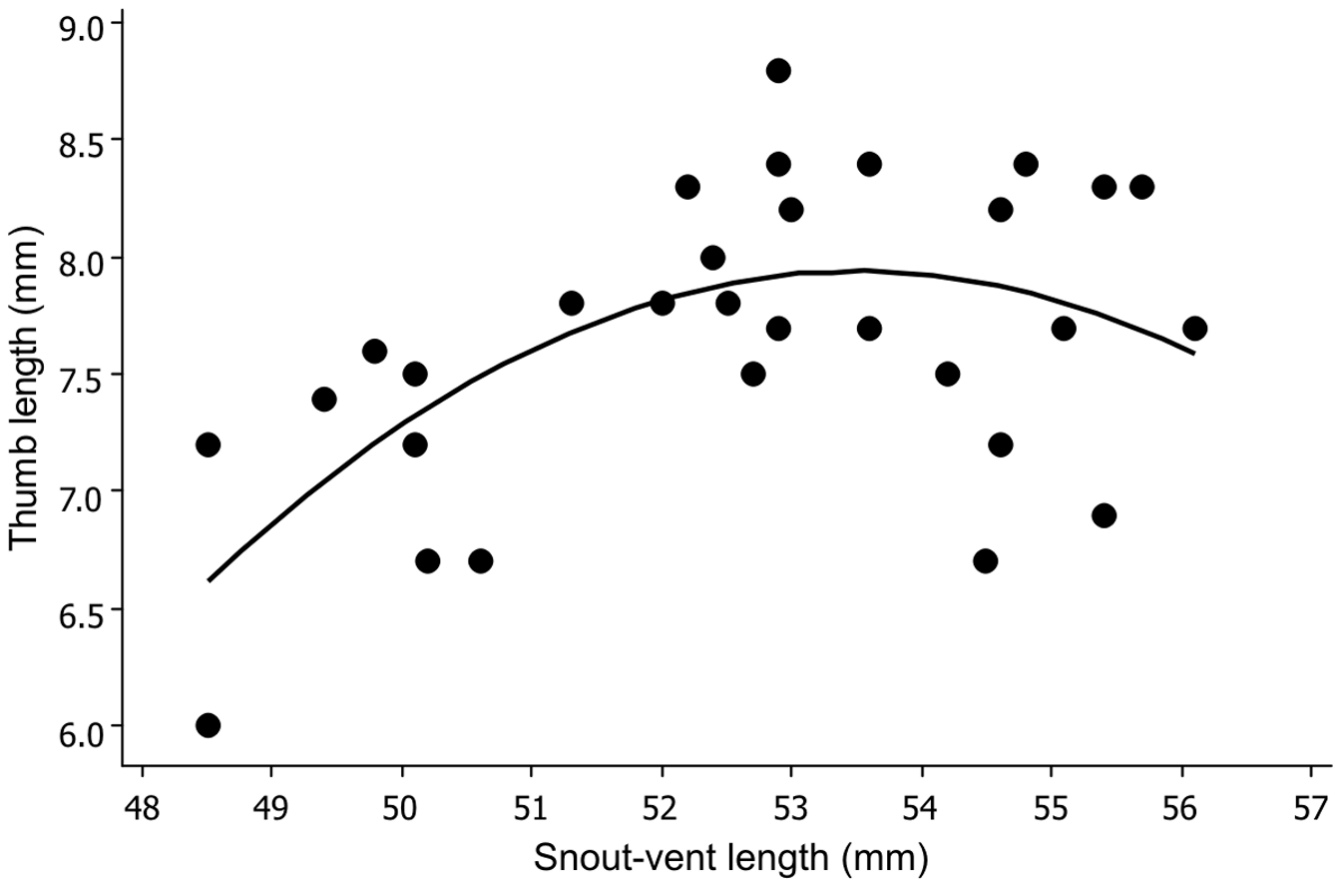
Relationship between thumb length and snout-vent length.

Sperm viability was relatively high across males with on average 91% (±1% SE) sperm within males ejaculates viable. The proportion of live sperm was uncorrelated with male morphological traits (*P*>0.30 for all traits). Thumb length showed a significant non-linear relationship with male snout-vent length, tapering off with increasing male body length (and age). Fitting the quadratic equation thumb length  = −142.8+5.6 (svl) - 0.053 (svl)^2^ increases *R* ^2^ from 0.19 to 0.34 compared to a linear relationship (polynomial *F*_2, 27_  = 7.0, *P* = 0.004; Figure 3).

## Discussion

Our study shows that male size per se did not significantly explain female mate preference, however intrinsic male siring success did. Females associate with males that have greater fertilization abilities but it remains unclear which traits females are using to detect this. There are a number of traits that were unmeasured in this study that have been shown to be used by discerning females such as call rate and call intensity [4], [5], [6], [7]. Longer thumbs just fell short of being significantly non-preferred by females and interestingly, we found that thumb size is a significant indicator of fertilization failure, with relatively longer thumbed males siring fewer offspring and the offspring that they did sire survived less well through early ontogeny. Thumb length is important for successful amplexus and has been shown to be under sexual selection in anuran amphibians [10], [25]. A positive relationship between male nuptial pad size and success in amplexing females has been shown for the Columbia Spotted Frogs (*Rana luteiventris*) [10]. However, the non-linear relationship between thumb length and body length in this study may capture some aspect of ageing; although there are two large (old) males with relatively short thumbs (Figure 3), most long-thumbed males are still the largest ones in terms of body size and most likely to be the oldest. The age-body size relationship of amphibians is well established [27], [28], [29], [30], and a negative relationship between sperm viability and male body size has been reported for the quacking frog *C. georgiana*, which may be a function of age or an alternative tactic of differential investment in spermatozoa by smaller-sized males [31]. Although we didn't detect any effect of SVL on fertilization success, if thumb length increases allometrically with age more than with body size, then this may explain its unexpected predictive power of fertilization success, via age-related DNA damage of spermatozoa that compromise fertilization. Alternatively, females may have chosen males based on some other unmeasured trait, such as call rate or call intensity which may be indicative of male age or male quality. DNA damaged sperm have been shown to have age-related effects in other taxa such as mice, bulls and humans [32], [33], [34], but further experimental work is needed to directly test if any relationship exists between age-related effects on sperm quality and fertilization success in amphibians.

In summary, we show that females prefer males that have high fertilization success. The only trait that we could link to high fertilization failure was relatively long thumbs in males (which just fell short of being significantly non-preferred in females). Long-thumbed males not only sired less offspring in competition with another male, but the offspring that they did sire also survived less well in early ontogeny. This work encourages more in-depth analysis of age-related male effects on sperm and DNA quality.

## Materials and Methods

### Collection of experimental animals

We collected adult frogs (*Rana arvalis*) from a single population at Viskafors, Sweden. Frogs were weighed (nearest 0.1 g), snout vent length (SVL) measured (nearest mm), and males assigned to one of two size categories, large (SVL >52 mm) or small (SVL <50 mm). While size is not a dichotomous trait in this species, the two size classes were chosen to increase the probability of detecting any size effects on female choice. Animals were held and experiments carried out at the University of Gothenburg. As required by the University of Gothenburg, ethics approval for the handling and housing of animals, and permission for the collection of animals, were sought and received by the Swedish central animal ethics committee (Permit 85-2005) and the Djurskddsmyndigheten (85-2005 which is a continuation of 326-2002).

### Female pre-copulatory mate choice: Experimental design

**Figure 4:**
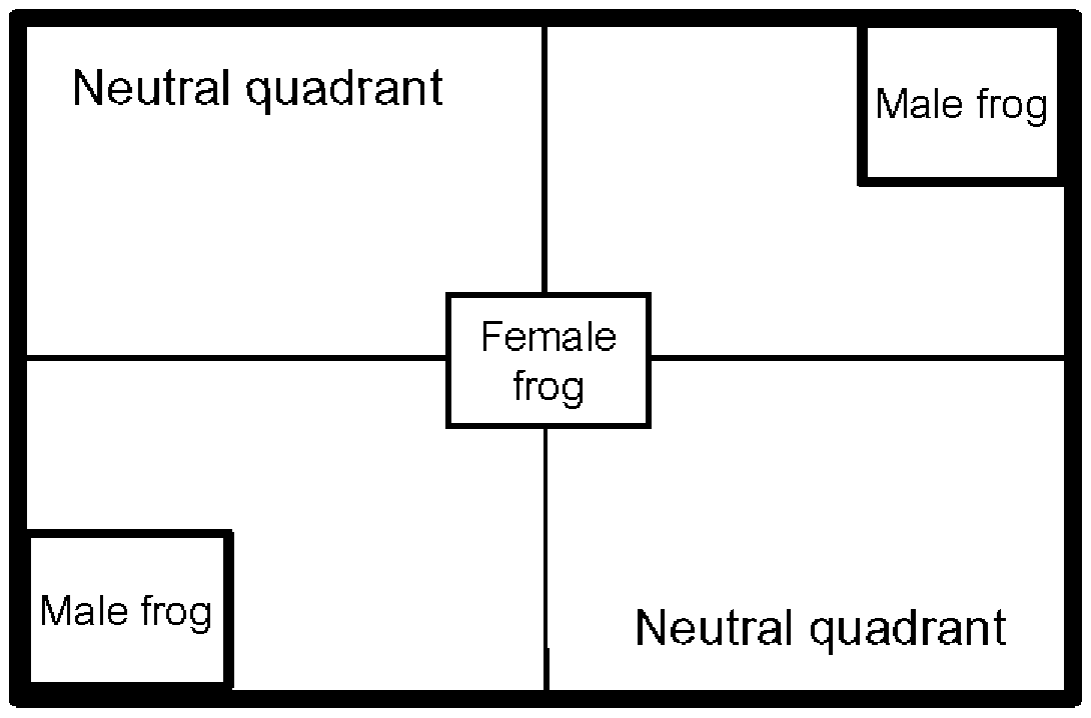
Diagrammatic representation of the arena used in mate choice trials.

For each mate choice trial a large and small male were randomly selected and placed in a plastic container with clear mesh sides. Containers were then placed at opposite corners of a larger mate choice arena (120 cm ×80 cm) filled with aged water (3 cm deep). A randomly selected female was placed equidistant between the two males and left for eight hours with no observations made during this period. We waited for this extended period before assessing mate choice so that all individuals could acclimatize to the mate choice arena, and so that females had sufficient time to assess males before making a choice. Females were scored after the eight hour period as having chosen a male if she was observed in a males quadrant. Females located in either of the two neutral quadrants were scored as not having shown a preference (Figure 4). A total of 22 mate choice trials were carried out and males or females were used only once. However, for three of these trials, females escaped from the mate choice arena and no data were recorded. For the remaining 19 trials, females were found in one of the two male's quadrants and no females were found within the neutral quadrants indicating that females were preferentially associating with one of the males.

### Fertilization and sperm competition experiments

The males and the female used in the mate choice trials were also used for subsequent fertilization and sperm competition experiments. We followed the protocol for artificial fertilization outlined in Sherman et al. [21], [22], [35]. The frogs were injected with approximately 150 µl per 10 g body weight of a 5 mg/100 ml luteinizing hormone releasing hormone (LHRH) in isotonic saline solution (0.9% NaCl). This induces ovulation within 24 h and sperm shedding within 1 h of injection in females and males, respectively. LHRH is now widely used to obtain viable sperm for experimentation without sacrificing study animals and has previously been used in this species to achieve high fertilization rates [36], [37]. The presence of viable sperm was confirmed by observation under a microscope at 200× magnification. Males were injected within two minutes of each other to ensure that longevity of sperm did not confound our experiments. The amount of sperm released by each male was determined using a Hawksley haemocytometer and varied from 9.1×10^6^ to 6.4×10^5^ sperm per ml. All sperm samples were diluted to equal concentrations (5×10^5^ sperm cells per ml) for use in the fertilization trials [38]. The proportion of viable sperm within each male's ejaculate was determined at the time of fertilization using Live/Dead® sperm viability kits (Molecular Probes)[39] and all sperm were used within the 1 h period after injection with LHRH. One milliliter of each male's sperm solution were mixed together and placed in a Petri dish. A control Petri dish for each male received sperm from only one male and was used to assess each male's ability to fertilize a female's eggs in the absence of sperm competition (i.e. non-competitive fertilizations). Eggs were stripped from each female by gently squeezing her abdomen and eggs were partitioned into each Petri dish (mean  = 79±3.9 eggs per dish). After three hours all eggs were transferred to a 750 ml container and held at a constant temperature of 20°C until hatching. Hatching success was determined as the number of tadpoles that successfully hatched from fertilized eggs.

### Paternity assignment

A total of 25–30 tadpoles per sperm competition trial (mean per trial ± SE, 25.4±0.84) were collected for the assignment of paternity. A toe clip from each adult was used for DNA extraction and the assignment of paternity. Genomic DNA was isolated using Qiagen DNAeasy Tissue Kit as per the manufactures instructions. Six microsatellite loci were used to assign paternity; RA11, RA13, RA14 (Sagvik unpublished data, Genbank Accsssion numbers: EU871712, EU871713 & EU871714); RlaCa41 [40] and WRA6-8 WRA1-22 [41]. Paternity was unambiguously assigned to all offspring (381 tadpoles) according to allele sharing between putative sires, dam and offspring. Genetic similarity among individuals was determined as the proportion shared alleles and calculated using GenAlex (V6) [42].

### Statistical analysis

#### (i) Female pre-copulatory mate choice

We performed a logistic regression (SAS, 9.1; Proc Logistic, binary logit link function) with choice of small male (1 =  chosen, 0 =  not chosen) as response variable, male difference in mass, arm length, thumb length and male difference in siring success in sperm competition as predictors. Predictor variables were backwards-eliminated at P<0.25. The significance of predictors were tested using log likelihood ratio tests.

#### (ii) Male siring success in sperm competition

We performed a multiple regression analysis with the difference in male siring success in sperm competition as the response variable, and the same set of predictors as described above (i). Difference in sperm viability between competing males was also included as an additional predictor in the model.

#### (iii) Hatching success

In this analysis we performed a multiple regression with the hatching success in controls. Again, we used the same set of predictors as described above (i) and (ii).

#### (iv) Male traits and sperm quality

In order to assess covariation between morphological traits and sperm quality, we looked for correlations between sperm viability and morphology traits (mass, arm length, thumb length).

#### (v) Genetic Similarity

Our index of genetic similarity was entered into the models above but was consistently non-significant and is not further reported on (*P*>0.25).
